# The impact of lamb diets containing either barley or corn on growth performance and carcass quality

**DOI:** 10.14202/vetworld.2021.1487-1491

**Published:** 2021-06-10

**Authors:** M. Ata, Belal S. Obeidat

**Affiliations:** 1 Department of Animal Production and Protection, Faculty of Agriculture, Jerash University, Jerash 26150, Jordan; 2 Department of Animal Production, Faculty of Agriculture, Jordan University of Science and Technology, Irbid 22110, Jordan

**Keywords:** Awassi lambs, barley, carcass, corn, performance

## Abstract

**Background and Aim:**

Grains, such as barley (BAR) and corn (CORN), are major energy sources for small ruminants. This study aimed to assess the impact of feeding either BAR or CORN-based diets on growth performance and carcass characteristics.

**Materials and Methods:**

Awassi male lambs, average body weight of 20.4±0.49 kg, were chosen randomly. Lambs were acclimated for 10 days and assigned to one of two diets (1) BAR and (2) CORN. Feeding continued for 70 days with 60 days of data collection. Daily intake was recorded. Measurements of body weight were taken starting from day 1 then once per week throughout the study period. On day 40, six lambs were randomly chosen from each group and placed in metabolism cages to assess digestibility and N balance. Lambs were slaughtered on the last day of the study to evaluate carcass characteristics and meat quality.

**Results:**

Neutral and acid detergent fiber and ether extract intake were greater (p≤0.05) for lambs fed the BAR diets. Nitrogen loss in feces tended to be greater (p=0.09) for the CORN diet. Eye muscle depth (mm) tended to be greater (p=0.07) for the BAR diet. Nutrient digestibility, daily weight gain, carcass characteristics, and meat quality were not different (p≥0.1) between diets.

**Conclusion:**

The results herein demonstrate that feeding BAR grain improved nutrient intake efficiency and consistency and did not affect weight gain and carcass traits. BAR-based diets might be a useful alternative to CORN for feeding growing lambs.

## Introduction

The sheep population in Jordan is estimated to be 3 million head [1]. Awassi is the predominant breed [2]. Awassi sheep are distributed in more than 30 countries due to their superior meat and milk production. The bred is considered transboundary and international [3]. The productivity of Awassi lambs depends on feed. Whole grains, such as barley (BAR) and corn (CORN), are the main ingredients in lamb diets since the availability of good quality forage in Jordan is limited by the harsh environment [4]. The nutritive value of BAR and CORN is reflected mainly in energy content; protein is considered of secondary importance [5]. In Jordan, CORN costs 50%-80% more than BAR. The latter is produced locally while CORN is imported. Consequently, using CORN in greater proportions in feed is less economical compared to feed formulated with high BAR content [6].

Mixing different grains containing varying amounts of degradable carbohydrate improve growth performance and efficiency in ruminants [7,8]. Grain ratios for improving microbial activity in ruminants rely on the management of carbohydrate and protein content [9]. Animal performance and ruminal starch fermentation were increased by combining slowly and rapidly fermenting grains compared to feed using single grains [10]. Further, starch and protein from BAR are fermented more quickly and efficiently in the rumen than CORN starch and protein [11]. BAR also stimulates energy and nitrogen release, thus improving the absorption of microbial nutrients. Accordingly, BAR might be a good replacement for protein sources that are used in ruminant diets [12].

The study hypothesis was that feeding whole grain-based diets (BAR vs. CORN) will improve lamb performance, carcass, and meat characteristics. This study aimed to investigate the effects of feeding either BAR or CORN on intake, nutrient digestibility, performance, and meat and carcass quality of Awassi lambs.

## Materials and Methods

### Ethical approval

This study was approved by Institutional Animal Care and Use Committee, Jordan University of Science and Technology (JUST).

### Study period and location

The study was conducted from January to April 2020 at the Agriculture Center for Research and Production of JUST. Samples collected during the study were analyzed at the Department of Animal Production Laboratories.

### Study procedures and sample analysis

Thirty Awassi male lambs, 20.4±0.49 kg average body weight, were assigned randomly into two groups using a completely randomized design. Lambs were fed either a BAR-based diet or a CORN-based diet (Table-1). Diets were prepared to provide fattening Awassi lambs with similar crude protein (CP) content (160 g/kg CP: Dry matter (DM) basis) [13,14]. Lambs individually housed in specially designed pens (1.5 m×0.75 m) and fed twice per day at 0900 and 1600 h with equal meals. Feeding continued for 70 days, following an acclimation and adaptation period of 10 days. Diets were offered every day to allow *ad libitum* intake. Uneaten feed was weighed throughout the study and stored at −20°C for analysis. Lambs were weighed on day 1 then weekly until the end of the experiment. On day 40, six animals were chosen randomly from each group and housed individually in metabolism cages (1.05 m×0.80 m) for evaluating digestibility. Five days of acclimation to cages were followed by 5 days of sample collection. Amounts of feed intake and refusal during the digestibility study were recorded and sampled for analysis. Collected feces were weighed daily. About 100 g/kg of feces was stored for subsequent analyses.

**Table-1:** Ingredients and chemical composition of diets containing either BAR or CORN grains fed to Awassi lambs.

Item	Diets	

	BAR	CORN
Ingredients (g/kg DM)		
BAR grain	500	0
CORN grain	0	500
Soybean meal	150	180
Alfalfa hay	110	170
Wheat straw	220	130
Salt	10.0	10.0
Limestone	9.0	9.0
Vitamin-mineral premix 1	1.0	1.0
Feed cost/ton (US$) 2	426	462
Nutrients (g/kg DM)		
Dry matter	90.8	90.4
Crude protein	160	160
Neutral detergent fiber	334	248
Acid detergent fiber	196	155
Ether extract	38.9	39.4

^1^Composition per kg contained (Vitamin A, 600,000 IU; Vitamin D3, 200,000 IU; Vitamin E, 75 mg, Vitamin K3, 200 mg; Vitamin B1, 100 mg; Vitamin B5, 500 mg; lysine 0.5%; DL-methionine, 0.15%; manganese oxide, 4000 mg; ferrous sulfate, 15,000 mg; zinc oxide, 7000; magnesium oxide, 4000 mg; potassium iodide, 80 mg; sodium selenite, 150 mg; copper sulfate, 100 mg; cobalt phosphate, 50 mg, dicalcium phosphate, 10,000 mg. ^2^Calculated based on the current prices. BAR=Barley, CORN=Corn, DM=Dry matter

Samples of feedstock and uneaten residual were dried at 55°C, weighed, and ground for further analysis. Feed, residual, and feces were analyzed for DM, CP, ether extract (EE), neutral detergent fiber (NDF), and acid detergent fiber (ADF). Analysis of samples used AOAC [15] procedures for DM with an air-forced oven set to 100°C for 24 h, CP (Kjeldahl procedure) and EE (Soxtec procedure, Soxtec System HT 1043 Extraction Unit, Tecator, Box 70, Hoganäs, Sweden) were also measured. Analytical procedures for NDF and ADF were performed according to Van Soest *et al*. [16] with adjustments for use in the fiber analyzer (Ankom2000) (Ankom Technology Cooperation, Fairport, NY, USA). Analysis of NDF used sodium sulfite and alpha-amylase (heat stable). NDF was then calculated using residual ash content.

### Slaughtering procedures, carcass, and meat quality evaluation

Slaughtering was performed at the Agriculture and Production Facilities Center at the end of 70 days. Lamb fasted body weight was recorded 18 h after the last meal. Lambs were slaughtered by qualified personnel at 0900 h as previously described by Obeidat [17]. Immediately after slaughter, weight was recorded as hot carcass weight. After chilling at 4°C for 24 h, cold carcass weight was recorded. Dressing percentage used cold carcass weight divided by fasted live weight. Non-edible parts of the carcass were removed and weighed immediately after slaughter. Linear dimensions and longissimus muscle measurements were recorded using chilled carcasses [17]. Carcasses were divided into four parts – shoulder, rack, loin, and leg cuts. Loin cuts were dissected to remove the longissimus muscle. Muscle samples were packed and stored in vacuum pack bags at −20°C for 2 weeks until evaluation.

Meat quality variables of pH, cooking water loss, water holding capacity (WHC), shear force values, and color (CIE L*a*b* coordinates) were measured. Frozen longissimus muscles were thawed at 4°C overnight using a chiller. Meat quality measurement used muscle tissue after cutting slices of exact thickness [18]. Color measurements used meat slices 15 mm thick with a colorimeter (12MM Aperture U 59730-30, Cole-Parameter International, Accuracy Microsensors Inc., Pittsford, NY, USA). Meat slices were distributed on a polystyrene tray and covered with a permeable film to allow access to oxygen for 2 h at 4°C. Meat slices 25 mm thick were used for cooking loss measurements. Slices were weighed, distributed into plastic bags, and cooked for 90 min using a water bath at 75°C. After cooking, slices were again weighed to calculate percentage water loss. Cooked slices were kept overnight at 4°C, and six cores of about 1-mm^3^ were cut from the slices to measure shear force values. A perpendicular to the direction of muscle fiber known as a Warner–Bratzler shear blade was used. pH was measured using pH meter after thawing meat samples. WHC used 5 g of raw meat chopped into tiny pieces and placed between two filter papers and two quartz plates. Tissues were squeezed under a 2500 g weight for 5 min to remove intracellular water. Meat samples were removed and weighed. WHC was calculated as WHC % = (initial weight−final weight)×100/initial weight.

### Statistical analysis

Data were analyzed using the MIXED procedure in SAS (version 8.1, 2000, SAS Inst. Inc., Cary, NC, USA). Fixed effects included only treatments for all data. Individual lambs were the random variable. Least square means were used to identify significance and differences among means at p≤0.05.

## Results

Nutrient intake improved for lambs consuming BAR (Table-2). DM and CP intake were similar (p=0.9) for the two experimental diets. Intakes of NDF and ADF were greater (p<0.05) and the intake of EE was lower (p<0.05) for lambs who consumed BAR compared to CORN.

**Table-2:** Effects of feeding either BAR or CORN grains on nutrient intakes of growing Awassi lambs.

Item	Diets			

	BAR (n=15)	CORN (n=15)	SEM	p-value
Nutrient intake				
Dry matter, g/days	1064	1066	40.8	0.9698
Crude protein, g/days	171	171	6.54	0.9887
Neutral detergent fiber, g/days	355	264	11.85	< 0.0001
Acid detergent fiber, g/days	208	165	7.13	0.0005
Ether extract, g/days	41.4	42.0	1.60	0.0005

BAR=Barley, CORN=Corn, SEM=Standard error of the mean

Neither BAR nor CORN diets affected nutrient digestibility or N balance measurements, except for N lost in feces (Table-3). A tendency (p<0.09) was noticed for greater N loss in lambs fed CORN compared to BAR. ADG and total weight gain were not affected by diet, even though initial weights tended to be greater (p=0.06) for the BAR group versus the CORN group (Table-4). Carcass and non-edible carcass characteristics were not affected (p>0.05) by diet (Table-5). Carcass linear dimensions were almost the same for the two groups, except for eye muscle depth. This parameter tended to be greater (p=0.07) for lambs fed BAR (Table-6). Meat quality was also not affected (p<0.05) between lambs from the two feeding groups (Table-7).

**Table-3:** Effects of feeding either BAR or CORN grains on nutrient digestibility and N balance of Awassi lambs.

Item	Diets			

	BAR (n=6)	CORN (n=6)	SEM	p-value
Digestibility				
DM	80.5	83.7	2.34	0.2427
CP	80.8	81.6	2.56	0.7690
NDF	66.2	65.7	4.48	0.9234
ADF	61.2	60.7	1.27	0.7890
EE	86.6	83.6	2.55	0.2314
N balance				
N intake, g/d	27.3	28.9	1.48	0.3126
N lost in feces, g/d	5.3	6.9	0.64	0.0962
N lost in urine, g/d	10.2	9.4	1.52	0.7216
Retained N, g/d	11.8	12.7	1.48	0.6866
N retention, %	44.1	43.9	5.51	0.9789

BAR=Barley, CORN=Corn, DM=Dry matter, CP=Crude protein, NDF=Neutral detergent fiber, ADF=Acid detergent fiber, EE=Ether extract

**Table-4:** Effects of feeding either BAR or CORN grains on growth performance of Awassi lambs.

Item	Diets			

	BAR (n=15)	CORN (n=15)	SEM	p-value
Initial weight, kg	20.7	20.2	0.49	0.0608
Final weight, kg	33.5	33.5	1.09	0.9830
Average daily gain, g	213	222	16.2	0.7045
Total gain, kg	12.8	13.3	0.97	0.7045

BAR=Barley, CORN=Corn, SEM=Standard error of the mean

**Table-5:** Effects of feeding either BAR or CORN grains on carcass, non-carcass components, carcass cut weights and percentages, and dissected loin of Awassi lambs.

Item	Diets			

	BAR (n=15)	CORN (n=15)	SEM	p-value
Fasting live weight (kg)	32.9	30.9	1.16	0.2500
Hot carcass weight (kg)	15.5	14.9	0.63	0.5321
Cold carcass weight (kg)	15.1	14.4	0.59	0.4178
Dressing percentage	45.7	46.8	0.70	0.2268
Non-carcass components (kg)	1.39	1.41	0.036	0.5351
Carcass cut weights (kg)	12.8	12.4	0.49	0.5741
Fat tail (kg)	1.79	1.72	0.136	0.7047
Loin weight (g)	1053	994	69.9	0.5594
Subcutaneous fat (g/100 g)	11.53	12.20	1.365	0.7336
Intermuscular fat (g/100 g)	2.88	2.71	0.395	0.7721
Total fat (g/100 g)	14.37	14.91	1.558	0.8120
Total meat (g/100 g)	58.24	56.67	1.373	0.4356
Total bone (g/100 g)	26.21	26.40	1.192	0.9140
Meat to bone ratio	2.27	2.25	0.127	0.9215
Meat to fat ratio	5.11	4.93	0.780	0.8798

BAR=Barley, CORN=Corn

**Table-6:** Effects of feeding either BAR or CORN grains on carcass leaner dimensions of Awassi lambs.

Item	Diets			

	BAR (n=15)	CORN (n=15)	SEM	p-value
Leg fat depth (*L3*) (mm)	3.50	3.57	0.458	0.9194
Tissue depth (*GR*) (mm)	9.47	8.73	0.519	0.3348
Rib fat depth (*J*) (mm)	2.84	2.50	0.397	0.5545
Eye muscle width (*A*) (mm)	49.9	48.8	0.8766	0.3955
Eye muscle depth (*B*) (mm)	21.19	19.73	0.5394	0.0713
Fat depth (*C*) (mm)	2.00	2.17	0.188	0.5407
Shoulder fat depth (*S2*) (mm)	2.03	1.77	0.250	0.4625

BAR=Barley, CORN=Corn, SEM=Standard error of the mean

**Table-7:** Effects of feeding either BAR or CORN grains on meat quality of Awassi lambs.

Item	Diets			

	BAR (n=15)	CORN (n=15)	SEM	p-value
pH	5.83	5.82	0.015	0.5730
Cooking loss (g/100 g)	38.4	38.3	0.92	0.8524
Water holding capacity (g/100 g)	23.7	24.9	1.23	0.5031
Shear force (kg/cm^2^)	9.0	9.6	0.51	0.3822
Color coordinates				
L* (whiteness)	35.1	43.2	5.71	0.3315
a* (redness)	2.47	2.45	0.157	0.8650
b* (yellowness)	17.2	16.9	0.68	0.7717

BAR=Barley, CORN=Corn, SEM=Standard error of the mean

## Discussion

We hypothesized that lambs fed whole grain-based diets (BAR and CORN) would display better nutrient digestibility, growth performance, and feed utilization. Diets were designed to provide similar CP content to compare grains at similar dietary DM levels. A positive effect was observed by adding high moisture CORN with whole-grain diets. Such diets improved feed efficiency and daily weight gain in ruminants [19]. CORN starch is less well digested in the rumen than starch from BAR. CORN starch digestibility might increase following processing, while digestibility of BAR is not affected [11].

Further, a noticeable finding in the previous studies is improvement in ruminal and total intestinal tract starch digestion after adding grains to the feed. Thus, feed utilization and efficiency were partly improved in the current study. These improvements were the result of integrating dietary energy and protein from grain-based formulated diets. The similarity in outcomes between diets might be due to insufficiently processed grains.

An improvement in NDF and ADF intake was observed with lambs fed BAR. Conversely, intake of EE was reduced. Boss and Bowman [20] reported similar findings for BAR-fed steers that showed greater feed efficiency compared with steers fed dry-rolled CORN. Other authors noticed no effect on nutrient intake after feeding BAR or CORN to ruminants [21,22]. In contrast, an improvement in nutrient intake for animals fed CORN-based diets compared to BAR is reported [7,10,23,24]. This increase in intake efficiency may reflect the processing method, which, in turn, affects its digestibility in the rumen. Moreover, higher nutrient intake in ruminants consuming CORN-based diets is explained by higher starch degradability of BAR compared to CORN. This finding suggests a change in ruminal pH resulting in decreasing cellulolytic bacteria counts and accompanying decreased digestibility of nutrients [25].

Nutrient’s digestibility, nitrogen balance, weight gain, and carcass quality were not affected by diet in the current study. Johnson *et al*. [7] observed an improvement in nutrient digestibility for steers consuming BAR versus CORN, though daily weight gain and body weight of steers did not differ. Conversely, final weight and daily weight gain were greater for lambs fed CORN versus BAR diets [10]. The authors suggest higher ruminal pH and better starch ruminal outflow [26] in ruminants consuming CORN- versus BAR-based diets.

Carcass characteristics were enhanced by consuming CORN-based diets, consistent with the previous studies [7,24,27]. In the current study, total meat and eye muscle depth were slightly improved in lambs fed BAR. The opposite result was reported by Petit [24] where total tissue depth was slightly enhanced by feeding CORN compared to BAR. A slight increment in body weight in the form of meat rather than fat or bone was observed after feeding with BAR compared to CORN.

## Conclusion

Feeding BAR improved nutrient intake efficiency and consistency for enhanced weight gain and carcass traits. BAR can be used to replace CORN grain in feed for growing lambs.

## Authors’ Contributions

BSO and MA: Designed, supervised, and drafted the manuscript. Both authors read and approved the final manuscript.
